# Origin of alkali-rich volcanic and alkali-poor intrusive carbonatites from a common parental magma

**DOI:** 10.1038/s41598-021-97014-y

**Published:** 2021-09-02

**Authors:** Ivan F. Chayka, Vadim S. Kamenetsky, Nikolay V. Vladykin, Alkiviadis Kontonikas-Charos, Ilya R. Prokopyev, Sergey Yu. Stepanov, Stepan P. Krasheninnikov

**Affiliations:** 1 Institute of Experimental Mineralogy, Russian Academy of Sciences, Akademika Osipyana Str., 4, Chernogolovka, Russia 142432; 2 V.S. Sobolev Institute of Geology and Mineralogy Siberian Branch of the Russian Academy of Sciences, Koptyuga prospekt., 3, Novosibirsk, Russia 630090; 3 Institute of Volcanology and Seismology, Far Eastern Branch of the Russian Academy of Sciences, Petropavlovsk-Kamchatsky, Russia 683000; 4 Vinogradov Institute of Geochemistry SB RAS, 1a Favorsky St., Irkutsk, Russia 664033; 5 Helmholtz-Zentrum Dresden-Rossendorf, Helmholtz Institute Freiberg for Resource Technology, Chemnitzer Str. 40, 09599 Freiberg, Germany; 6 School of Chemical Engineering and Advanced Materials, The University of Adelaide, Adelaide, SA 5005 Australia; 7 Department of Geology and Geophysics, Novosibirsk State University, Novosibirsk, Russia 630190; 8 Federal State Budgetary Insitution “A.P.Karpinsky Russian Geological Research Institute” (FGBU “VSEGEI”), St. Petersburg, Russia 199106; 9 Vernadsky Institute of Geochemistry and Analytical Chemistry, Russian Academy of Sciences, Kosygin St. 19, Moscow, Russia 119991

**Keywords:** Geochemistry, Geology, Mineralogy, Petrology, Volcanology

## Abstract

The discrepancy between Na-rich compositions of modern carbonatitic lavas (Oldoinyo Lengai volcano) and alkali-poor ancient carbonatites remains a topical problem in petrology. Although both are supposedly considered to originate via fractional crystallization of a “common parent” alkali-bearing Ca-carbonatitic magma, there is a significant compositional gap between the Oldoinyo Lengai carbonatites and all other natural compositions reported (including melt inclusions in carbonatitic minerals). In an attempt to resolve this, we investigate the petrogenesis of Ca-carbonatites from two occurrences (Guli, Northern Siberia and Tagna, Southern Siberia), focusing on mineral textures and alkali-rich multiphase primary inclusions hosted within apatite and magnetite. Apatite-hosted inclusions are interpreted as trapped melts at an early magmatic stage, whereas inclusions in magnetite represent proxies for the intercumulus environment. Melts obtained by heating and quenching the inclusions, show a progressive increase in alkali concentrations transitioning from moderately alkaline Ca-carbonatites through to the “calcite CaCO_3_ + melt = nyerereite (Na,K)_2_Ca_2_(CO_3_)_3_” peritectic, and finally towards Oldoinyo Lengai lava compositions. These results give novel empirical evidence supporting the view that Na-carbonatitic melts, similar to those of the Oldoinyo Lengai, may form via fractionation of a moderately alkaline Ca-carbonatitic melt, and therefore provide the “missing piece” in the puzzle of the Na-carbonatite’s origin. In addition, we conclude that the compositions of the Guli and Tagna carbonatites had alkali-rich primary magmatic compositions, but were subsequently altered by replacement of alkaline assemblages by calcite and dolomite.

## Introduction

Carbonatites are carbonate-dominated (> 50% modal primary carbonates) igneous rocks, which occur worldwide and are typically found within intraplate alkaline magmatic provinces, varying in age from Archean to the present^1,2^. Although carbonatites are exotic and generally occur in small volumes, they are an important global source of critical elements^1,3^. They also provide valuable insights into compositions of the mantle sources, melting processes and carbon geochemical cycles on Earth^1,4,5^. Modern petrogenetic models widely acknowledge that most carbonatites crystallize directly from carbonate-dominated melts, which form via magma differentiation and immiscibility from precursor carbonated alkaline silicate melts or via ultra-low degree melting of a carbonated mantle source^1,6–9^.

The compositional gap between Na-rich compositions of the only modern carbonatitic lavas (Oldoinyo Lengai volcano) and ancient alkali-poor intrusive carbonatites has strongly influenced petrogenetic concepts for carbonatites. Such a uniqueness was initially thought to be attributed to assimilation of Na-evaporites in the carbonatite magma at Oldoinyo Lengai^10^; however, this was later contradicted by their mantle-like isotopic composition^11^. On the other hand, the presence of alkaline assemblages (carbonates, sulfates, phosphates and halides) as inclusions within primary carbonatite mineral phases worldwide indicates the universal involvement of Na-carbonatitic melts, and thus a petrogenetic link between the alkaline and alkali-poor compositions^12–25^. An assumption that a moderately alkaline Ca-carbonatitic melt may be a common parent for both Na-rich and alkali-poor carbonatites is acknowledged as a feasible solution for this conundrum^15,26^. However, there has been a lack of empirical insights into: (1) the behavior and potential loss of alkalis during formation of intrusive carbonatites, and (2) the possibility of Na-carbonatites being produced through fractionation of such a “common parent”.

In this study, we address this problem from the perspective of intrusive carbonatites by examining the Guli and Tagna (Bol’shaya Tagna) intrusions (Siberia, Russia), which are texturally and mineralogically representative of apatite-magnetite calcic carbonatites from a number of occurrences worldwide (e.g. Kovdor, Arbarastakh, Oka and Vuorijarvi)^14,27^^’ data^. In particular, we investigate their textural features and multiphase inclusions hosted in apatite and magnetite to unravel their petrogenetic history, including parental melts, and determine whether the latter may form the missing link between intrusive alkali-poor and extrusive Na-rich carbonatites.

### Geological background

**Figure 1:**
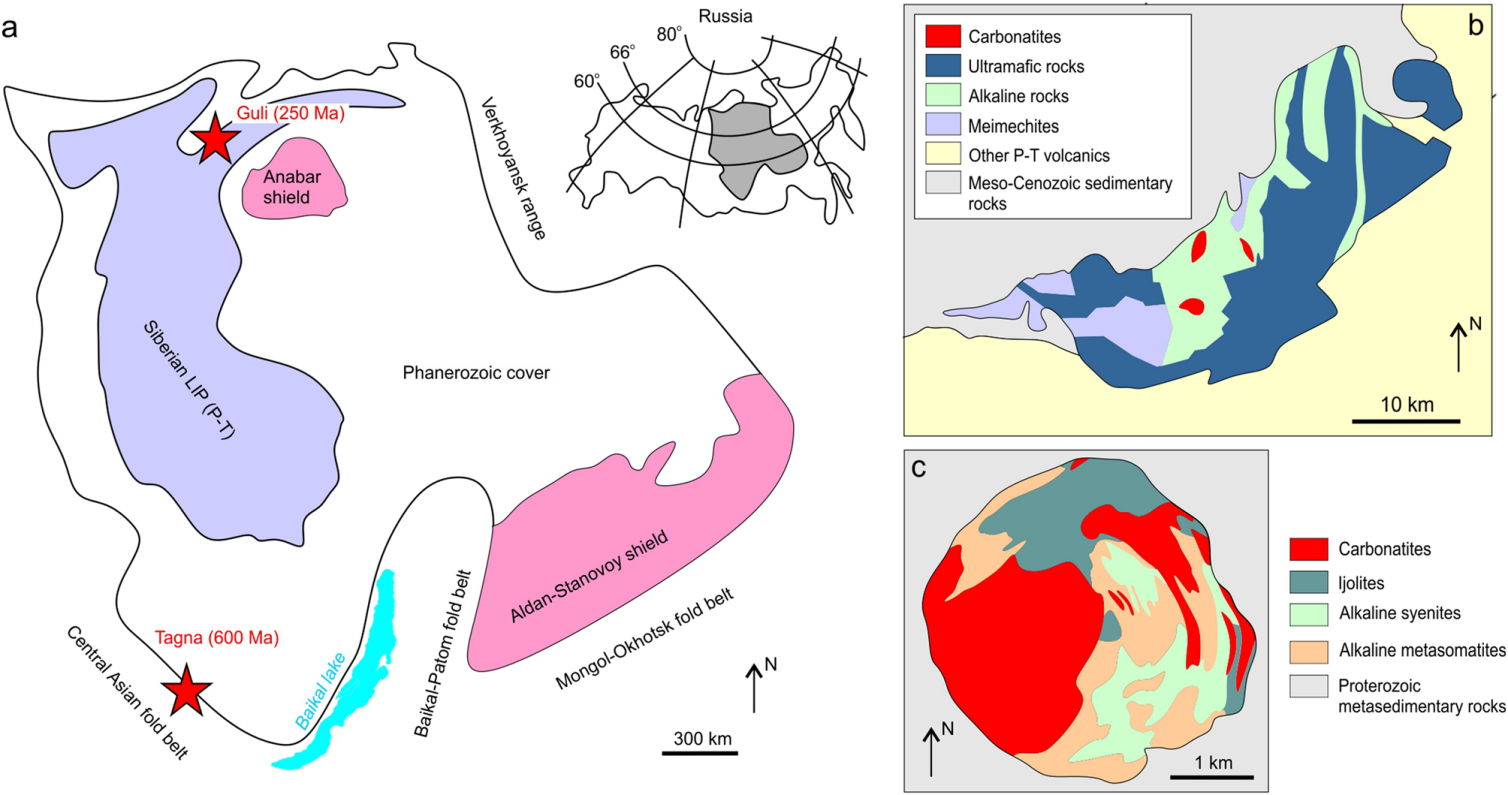
Geological layout of the studied massifs. (**a**) location of the Guli and Tagna massifs within the Siberian platform (modified after^33,34^); (**b**) Geological scheme of the Guli massif (modified and simplified after^29^); (**c**) Geological scheme of the Tagna massif (modified and simplified after^31,32^). The figure was created using Corel Draw X4 software https://www.coreldraw.com/en/pages/coreldraw-x4/.

Both studied occurrences are located at marginal parts of the Siberian platform. The Guli massif (250.8 ± 1.2 Ma^28^, Northern Siberia), is found within the northeastern area of the Triassic Siberian Large Igneous Province (LIP) (Fig. 1a). It has an irregular shape and occupies an area of 35 × 45 km. Compositionally, the Guli massif mainly comprises ultramafic and alkaline rocks (dunites, peridotites, nepheline syenites) emplaced within the Permian–Triassic volcanics. Carbonatites are a minor occurrence, forming several discordant plug-like bodies within the central part of the massif (Fig. 1b)^29^. The Tagna massif (~ 600 Ma^30^) is located at the southwestern margin of the Siberian platform in the Eastern Sayan alkaline carbonatite-ultramafic province (Fig. 1a). It is roundish and forms a deformed zoned structure with lithologies varying from ijolite-melteigites, nepheline and K-feldspar syenite to alkaline metasomatic rocks, alnöites and carbonatites^31,32^ (Fig. 1c).

## Methods

### Specimen preparation and routine analysis

Samples of polished thin sections and rock chips were prepared for optical microscopy and scanning electron microscopy (SEM) to determine mineral textures and relationships. Mineral separates of apatite and magnetite allowed for the compositions of multiphase inclusions and semi-quantitative analysis of alkaline carbonates to be measured using SEM EDS analysis. Back scattered electron (BSE) and secondary electron (SE) photomicrographs, and EDS analyses were carried out on the following equipment: (1) Tescan Mira 3 LMU with Oxford INCA Energy XMax 80 detector for EDS analysis and (2) Jeol JSM 1650 LV (Analytical Center for Multi-Elemental and Isotope Research Siberian Branch, Russian Academy of Science, IGM SB RAS, Novosibirsk) (3) Tescan VEGA-II XMU INCA Energy 450 (TESCAN, Brno, Czech Republic) (EDS mode, 20 kV, 190 pA, 180 nm beam diameter, excitation zone of 3–4 μm) (IEM RAS, Chernogolovka, Russia) and (4) Hitachi SU-70 Schottky field emission SEM fitted with Oxford INCA Energy XMax 80 silicon drift detector EDS system (University of Tasmania). To avoid dissolution of water-soluble minerals within the inclusions, grinding and polishing were performed using water-free lubricants (kerosene, hexane and WD-40 oil). Raman spectroscopy (LabRam HR800 Horiba Jobin Yvon spectrometer, equipped with an optical microscope Olympus BX41, IGM SB RAS) was performed on both exposed and unexposed apatite-hosted inclusions as well as on exposed magnetite-hosted inclusions. The 514.5 nm Ar + laser line was used for spectral excitation. The well-known RRUFF (http://rruff.info) database was used to identify the solid phases. Chemical composition of olivine and apatite was determined by electron probe microanalysis with wavelength dispersive X-ray spectroscopy (WDS) on equipment JEOL JXA-8320 and JEOL JXA-8100 microprobes at the AC IGM SB RAS. Natural mineral compositions were used as standards and were analyzed every 30–50 analyses. Chemical analysis of calcite has been performed in EDS quantitative analysis mode Hitachi SU-70 Schottky field emission SEM fitted with Oxford INCA Energy XMax 80 silicon drift detector EDS system (University of Tasmania).

### Heating/cooling experiments and analysis of quenched melts/inclusions

For observed heating/cooling experiments grains of selected apatite were separated from crushed samples and double-polished. The experiments were carried out on Linkam THMSG-600 and TC-1500^35^ stages equipped with an automatic software temperature control and an optical Olympus BX41 microscope. The heating was performed in an atmosphere of purified argon.

To obtain a statistically representative dataset of the experimentally produced melts in the inclusions and to determine phase compositions of the partially-homogenized inclusions, we performed blind heating-quenching experiments on the mineral separates of apatite and magnetite. This experiment additionally allowed for observation of phase transformations in the magnetite-hosted inclusions at different temperatures and to estimate the temperature range of their (partial) homogenization. For the experiments, the mineral separates were sealed into loosely closed Pt ampoules. To prevent oxidation of magnetite at high temperatures by atmospheric oxygen, pure carbon (2 µm diamond powder) was added as a redox buffer to the magnetite separates. As carbonate melts do not form homogeneous glass and crystallize almost instantaneously, we aimed to cool the mineral separates as rapidly as possible. However, as this may lead to fracturing of the hosts and inclusion leakage, we performed experiments both in a muffle furnace SNOL 12/1300 (GEOKHI RAS) with subsequent manual placing of the ampoules into water (cooling time 5–10 s), and in a custom made vertical pipe furnace (IEM RAS) (Supplementary Fig. 6) with an ampoule dropped into water via cutting of the hanging wire (cooling time < 2 s). The pipe furnace consisted of a quartz glass pipe, wired by a spiral heater (kanthal FeCrAl alloy), kaolin wool wrapping and the outer tube (quartz glass) (Supplementary Fig. 6b). The temperature control system comprised a type-K thermocouple, placed within the pipe, a calibrated electronic thermometer (1 °C accuracy), which automatically switched off the heater when the pre-set temperature was reached, and a manual varistor, which allowed for gradual temperature increase (Supplementary Fig. 6a). Based on data of the observed homogenization experiments, apatite was quenched at 700, 800 and 900 °C. Magnetite was quenched at 600, 700, 800, 900 and 1000 °C, based on high-T spinel exsolutions^36^, previously reported data on inclusions in carbonatite-hosted refractory minerals^15,17,37^ and homogenization temperatures of the apatite-hosted inclusions. Heating speeds were 2 °C/min in the muffle furnace and 30–50 °C/min in the vertical pipe furnace. To provide sufficient phase equilibration within the inclusions, the run temperatures were sustained for 10 min in each experiment, after which the ampoules with separates were cooled.

After heating, the exposed and polished inclusions were examined using SEM EDS analysis, performed on Tescan Mira 3 LMU with Oxford INCA Energy XMax 80 detector for EDS analysis, Jeol JSM 1650 LV (Analytical Center for Multi-Elemental and Isotope Research Siberian Branch, Russian Academy of Science, IGM SB RAS, Novosibirsk) and Tescan VEGA-II XMU INCA Energy 450 (TESCAN, Brno, Czech Republic) (EDS mode, 20 kV, 140 pA, 180 nm beam diameter, excitation zone of 3–4 μm) (IEM RAS, Chernogolovka, Russia). Quantitative analysis of the experimentally obtained and quenched carbonate melts in the inclusions was carried out using the same SEM EDS equipment. Electron microprobe analysis was insufficient for this purpose due to rapid diffusion-driven loss of Na from the analyzing volume at high currents^38^, whereas SEM EDS analysis has been proved to provide reliable numeric data for elements which concentration exceeds 1 wt%^39^. To obtain more representative data and minimize Na loss, the signal was acquired on areas from 3 × 3 to 10 × 10 µm. Acquisition time was dependent on Na loss during the analysis, which was real-time monitored with the acquisition being terminated when the decrease of Na (difference between initial signal of Na and its current intensity) reached 2σ error. Average acquisition time thus varied between 15 and 30 s with analytical errors varying from 0.1 to 0.3% for Na and 0.1 to 0.2% for the other elements. After the analysis, each spectrum was processed manually in the Aztec (Oxford Instruments Nanotechnology Tools Ltd) software to include all elements with detectable peaks and exclude analytical artefacts. Detection limits for the majority of elements were around 0.2 wt%. Spot analyses of the quenched carbonate melts inside inclusions revealed considerable compositional variations, especially in Na and Ca concentrations. However, area analyses of cryptocrystalline aggregations were considered in order to minimize uncertainties and heterogeneity. Possible uncertainties derived from the surface irregularities or signals from neighboring mineral phases (e.g. host apatite, magnetite and Mg–Al oxides and hydroxides within magnetite) were dealt with by calculating numerical relationships between Ca, Na and K: Na_2_O/CaO and K_2_O/CaO ratios, as well as proportions of calculated CaCO_3_, Na_2_CO_3_ and K_2_CO_3_ molar values, instead of their measured absolute concentrations.

## Results

### Petrography and mineral relationships

**Figure 2:**
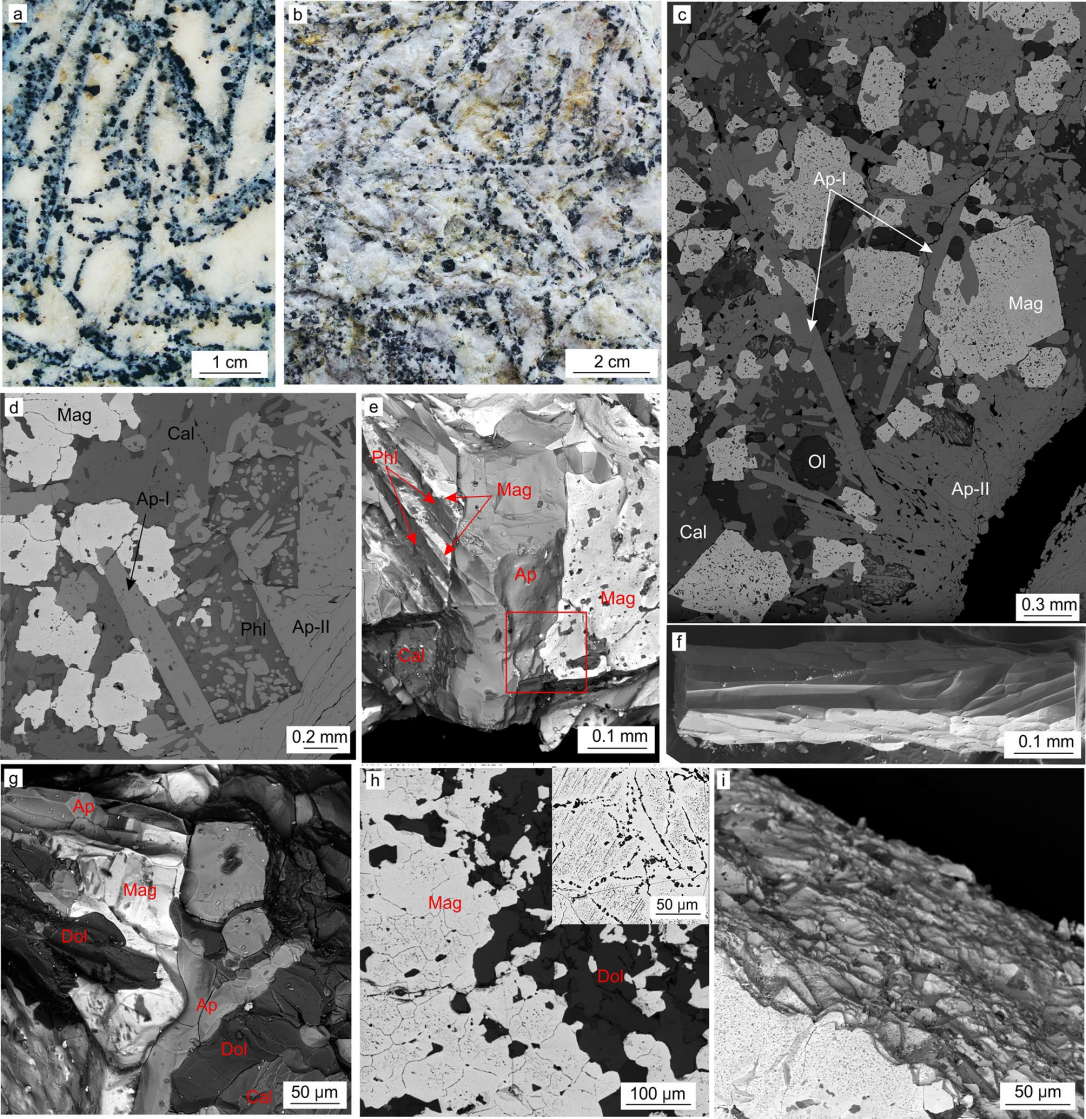
Textural features and mineral relationships of the rocks investigated. (**a**) carbonatite, Guli massif; (**b**) carbonatite, Tagna massif; (**c**) phase relationships, Guli carbonatite (BSE-image); (**d**) phase relationships, Tagna carbonatite (BSE-image); (**e**) apatite-magnetite intergrowth (BSE-image), outlined are intricate apatite-magnetite relationships; (**f**) apatite-II crystal, Guli carbonatite (SE image); (**g**) apatite-magnetite intergrowth, Guli massif (BSE-image); (**h**) multi-domain structure of magnetite, Tagna carbonatite, (BSE-image), corner inset—domains in magnetite grain bordered by trails of spinel inclusions, Guli carbonatite (BSE-image); (**i**) grain surface of a magnetite crystal, Guli carbonatite (BSE-image). Abbreviations: Ap—apatite, Cal—calcite, Phl—phlogopite, Ol—olivine. The figure was created by compilation of SEM photos using Corel Draw X4 software https://www.coreldraw.com/en/pages/coreldraw-x4/.

Hand specimens of both Guli and Tagna carbonatite samples display textural patterns with typical linear segregations of apatite and magnetite, settled within a calcite-dominated matrix (Fig. 2a,b). Anhedral granular grains of calcite predominate over lesser dolomite, which forms inclusions in calcite or fills interstices between calcite grains (Supplementary Figs. 1, 2). Apatite forms sheaf-like aggregations comprising two distinct morphologies: medium-grained euhedral prismatic crystals (apatite-I), and fine-grained needle-like crystals forming complex intergrowths with neighboring phases (apatite-II) (Fig. 2c–f; Supplementary Figs. 1, 2). Magnetite occurs as irregular and subhedral grains, typically displaying domains surrounded by trails of spinel exsolution and multiphase inclusions (Fig. 2c–e, g–i). Outer parts of magnetite grains generally contain dense disseminated inclusions of spinel, rock-forming minerals and alkaline multiphase inclusions. Other minerals within the samples include olivine, micas (mainly phlogopite), glagolevite, thorianite, perovskite, burbankite and pyrochlore-group minerals.

Rock-forming minerals (calcite, apatite and olivine) have relatively narrow ranges of chemical compositions (Supplementary Tables 1–3), and do not show any correlation between morphological features and chemistry. Apatite has moderate SrO (0.3–0.4 wt.%), LREE (La_2_O_3_ + Ce_2_O_3_ + Nd_2_O_3_ 0.3–0.45 wt. %) and F (1–1.3 wt.%) contents and contains only traces of Cl (< 0.04 wt.%) and SO_3_ (< 0.06 wt.%). Calcite contains 0.2–1.2 (average 0.6) wt. % of MgO and 0.3–1.2 (average 0.7) wt. % of SrO. Olivine is characterized by an extremely high Fo number (97–98 mol %), moderate MnO (0.2–0.3 wt.%), low CaO contents (0.05–0.09 wt. %) and is almost free of other admixtures. Due to dense and crowded spinel micro-inclusions, magnetite was not suitable for microprobe chemical analysis.

### Multiphase inclusions hosted in apatite and magnetite

**Figure 3:**
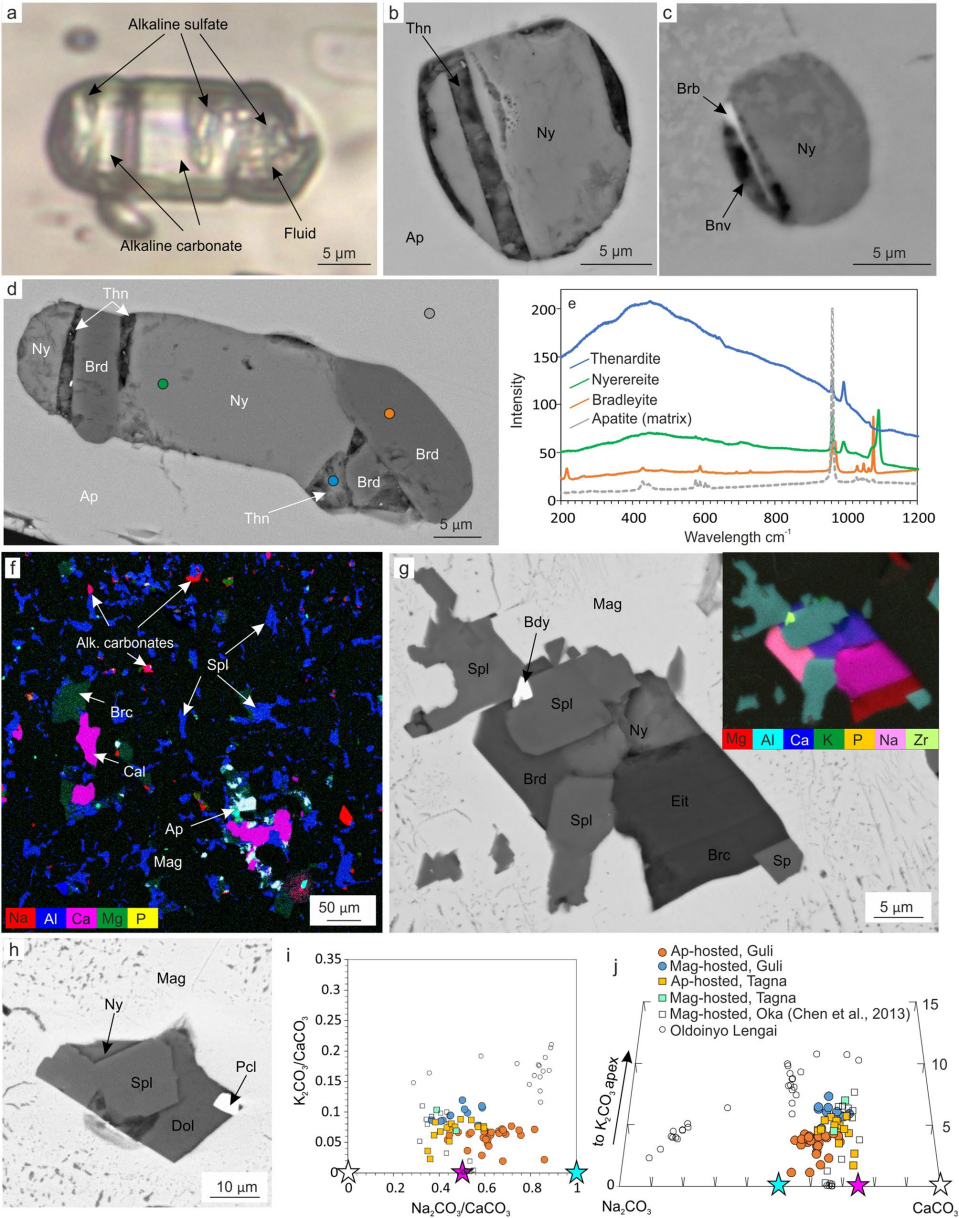
Multiphase inclusions. (**a**) apatite-I-hosted inclusion, Guli carbonatite (transmitted light); (**b**, **c**) BSE images of typical apatite-I-hosted inclusions from Guli and Tagna carbonatites, correspondingly; (**d**) apatite-hosted inclusion, Guli carbonatite (BSE image); (**e**) Raman spectra of mineral phases from apatite-hosted inclusions (analysis points are highlighted on the (**d**) panel); (**f**) inclusions in magnetite, Guli carbonatite (EDS elemental map) (**g**) magnetite-hosted inclusion, Guli carbonatite (BSE-image) with the EDS map on the inset; (**h**) magnetite-hosted inclusion, Tagna massif; (**i**) and (**j**)—variation diagrams with plotted compositions of the alkaline carbonates, enclosed in apatite and magnetite, and carbonates from magnetite-hosted inclusions of the Oka carbonatite^14^, alkaline carbonates from the Oldoinyo Lengai lavas, and stoichiometric compositions of calcite (white star), shortite (pink star) and nyerereite (blue star). Abbreviations: Ny—nyerereite, Thn—thénardite, Brb—burbankite, Bnv—bubnovaite, Brd—bradleyite, Ap—apatite, Spl—spinel, Mag—magnetite, Cal—calcite, Brc—brucite, Bdy—baddeleyite, Eit—eitelite, Dol—dolomite, Pcl—pyrochlore. Data for the Oldoinyo Lengai carbonatite are from the GeoRoc database (accessed 10 March 2021). The figure was created using Corel Draw X4 software https://www.coreldraw.com/en/pages/coreldraw-x4/, the plots were pre-designed in OpenOffice Calc (Apache OpenOffice 4.1.10) https://blogs.apache.org/OOo/entry/announcing-apache-openoffice-4-16.

In both carbonatite samples, primary inclusions are abundant in apatite-I, typically occurring as clusters elongated along the {001} axis, and their morphology is influenced by the host apatite. These inclusions can be divided into three groups: (1) fluid inclusions with minor chlorides and hydrous silicates, (2) calcite or dolomite-calcite inclusions and (3) multiphase inclusions, dominated by alkaline minerals, with minor fluid (see Supplementary Table 4 for the full mineralogical list and ideal chemical formulas). Our study focuses on the latter group of inclusions, which are dominated by SO_4_-bearing alkaline carbonates, ranging from shortite to nyerereite-fairchildite solid solution (Fig. 3a–d, Supplementary Table 5). Alkaline sulfate phases mainly include thénardite and bubnovaite, and subordinate aphthitalite (Fig. 3b–e). Calcite, burbankite and bradleyite are also common (Supplementary Table 4, Fig. 3b–e), whereas silicates such as glagolevite are rare.

Several groups of inclusions are also observed within magnetite from both carbonatites (Fig. 3f), such as: (1) spinel exsolution; (2) calcite and dolomite-calcite, and (3) alkali-rich multiphase inclusions (Fig. 3g,h). The latter are abundant in magnetite from the Guli carbonatite, and occasionally found within magnetite from the Tagna carbonatite. Spinel is typically present in each group of inclusions (Fig. 3f). Magnetite-hosted inclusions comprise more sodic minerals (eitelite, bradleyite, and lesser sulfates) than apatite-hosted inclusions (Supplementary Table 4). In addition, alkaline carbonates found in magnetite typically have lower S content and higher proportions of fairchildite K_2_Ca(CO_3_)_2_ (Fig. 3i,j; Supplementary Table 5). Inclusions of Mg and Al oxides and hydroxides are also unique to magnetite. Notably, the compositions of apatite- and magnetite-hosted alkaline carbonate inclusions are similar in both the Guli and Tagna carbonatites (Fig. 3i,j; Supplementary Table 4; Supplementary Table 5).

### Heating experiments

Observed heating and blind heating-quenching experiments were performed on the apatite- and magnetite-hosted multiphase inclusions (see “Methods” for the experimental procedures). In apatite-hosted fluid inclusions, dissolution of salt phases took place between 150 and 300 °C, followed by the decrepitation of all fluid inclusions between 300 and 500 °C. Melting of the multiphase inclusions began at temperatures ~ 400 °C, with sulfate minerals transforming first (Supplementary Fig. 3). Most of the inclusions decrepitated and leaked at temperatures between 400 and 700 °C (Supplementary Fig. 3). Those that survived became partially homogeneous (melt + gas bubble) in the temperature range 720–800 °C (e.g. Supplementary Fig. 4). Notably, gas bubbles in the inclusions did not dissolve or shrink significantly up to 1000 °C, which was the highest temperature attained.

**Figure 4:**
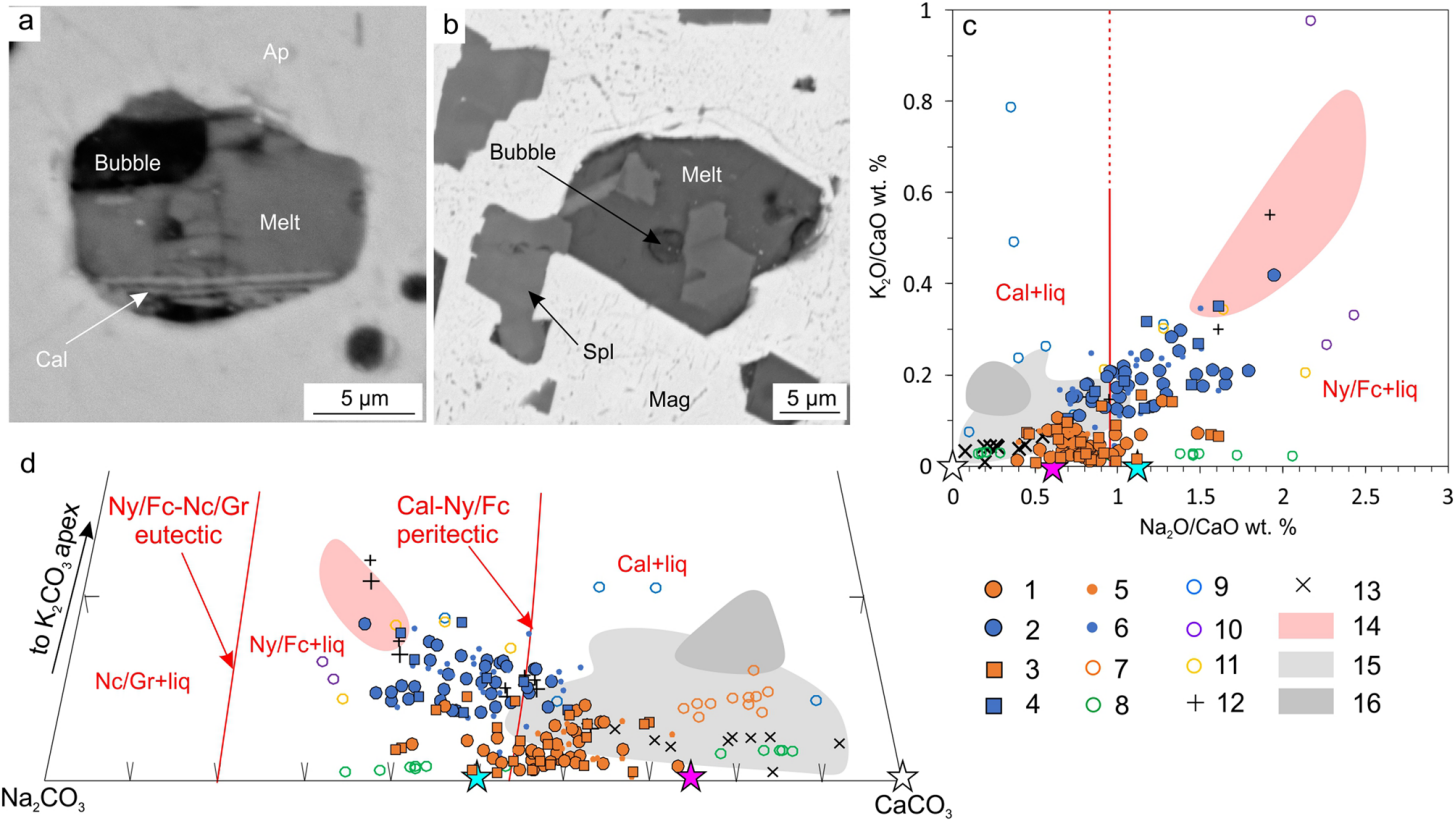
Images and geochemical data of heated inclusions. (**a**) apatite-I-hosted inclusion, quenched at 700 °C, Guli massif (BSE-image); (**b**) magnetite-hosted inclusion, quenched at 600 °C, Guli massif (BSE-image); (**c, d**) chemical variation diagrams of quenched inclusion compositions plotted with other inclusion and melt data from well-known carbonatites elsewhere. Legend: (1)—apatite-I-hosted inclusions, Guli massif, 700 and 800 °C (2)—magnetite-hosted inclusions, Guli massif, 700 and 800 °C (3)—apatite-hosted inclusions, Tagna massif, 700 and 800 °C (4)—magnetite-hosted inclusions, Tagna massif, 700 °C (5)—apatite-I-hosted inclusions, Guli massif, 900 °C; (6)—magnetite-hosted inclusions, Guli massif, 800–1000 °C (7)—homogenized (hereinafter all melt inclusions, mentioned in the caption, are experimentally homogenized) olivine- and magnetite-hosted inclusions, Belaya Zima massif^12^; (8)—melilite-hosted inclusions, Gardiner massif^20^; (9)—inclusions from carbonatite minerals of the Krestovsky massif^37^; (10)—fluorite-hosted inclusions, Tagna massif^13^; (11)—nepheline-hosted inclusions, Oldoinyo Lengai tephra^40^; (12)—experimental carbonatitic melt in equilibrium with peralkaline nephelinitic melt^41^; (13)—experimental carbonatitic melt in equilibrium with high-Ca nephelinitic melt^42^; (14)—bulk compositions of fresh Na-carbonatite lavas, Oldoinyo Lengai (GeoRoc database, accessed 21 March 2021); (15)—magnetite-hosted inclusions, effusive Ca-carbonatite, Kerimasi volcano^15^; (16) – perovskite-hosted inclusions, nephelinite, Kerimasi volcano^16^. Experimentally determined thermodynamic fields and univariant curves are outlined in red on (c) and (d)^26^. White, pink and blue stars represent stoichiometric compositions of calcite, shortite and nyerereite, respectively. Abbreviations: Cal—calcite, Spl—spinel, Mag—magnetite, liq—liquid, Nc—Na-carbonate, Fc—fairchildite, Ny—nyerereite, Gr—gregoryite. The figure was created using Corel Draw X4 software https://www.coreldraw.com/en/pages/coreldraw-x4/, the plots were pre-designed in OpenOffice Calc (Apache OpenOffice 4.1.10) https://blogs.apache.org/OOo/entry/announcing-apache-openoffice-4-16.

Apatite-hosted inclusions, exposed and examined by SEM after blind heating-quenching experiments (700, 800 and 900 °C), also showed that the complete melting occurred largely within 700–800 °C interval. Upon being rapidly cooled from these temperatures, the inclusions comprised cryptocrystalline sulfate-carbonate aggregates (formerly melt), a gas bubble, occasional relics of unmolten sulfates and thin crystals of calcite and hydrous silicates (newly formed quench phases) (Fig. 4a, Supplementary Fig. 5a).

Partial homogenization of the magnetite-hosted multiphase inclusions, according to the results of blind heating-quenching experiments, took place between 600 and 700 °C. Typical run products were homogeneous cryptocrystalline aggregates with occasional calcite and bradleyite, (which were slightly more abundant in the 600 °C experiments than at higher temperatures) brucite, apatite, micas and a gas bubble. Large grains of spinel, apatite and pyrochlore remained intact up to 1000 °C (Fig. 4b, Supplementary Fig. 5b-d). Importantly, in certain inclusions there were distinct “spinifex-like” quench structures, composed by thin crystals of nyerereite, which shows that the melts were nyerereite-saturated in contrast with calcite-saturated melts in the apatite-hosted inclusions (Supplementary Fig. 5b).

Compositions of quenched melts in apatite-hosted inclusions (Supplementary Table 6) correspond to a sulfate-rich alkaline carbonate magma with alkalinity concentrations similar to liquids found in melt inclusions from the Kerimasi volcano (Fig. 4c, d)^15,16^. However, melts from the magnetite-hosted inclusions were substantially more alkaline than those of the apatite-hosted inclusions (Supplementary Table 6; Fig. 4c,d). Their anomalously high alkalinity has never been reported from any known composition of melt inclusions from carbonatites worldwide (aside from three inclusions found in Tagna fluorite^13^). Interestingly though, they appeared to trend towards the bulk compositions of the Oldoinyo Lengai lavas (Fig. 4c,d).

## Discussion

### Interpretation of the experimental results

**Figure 5:**
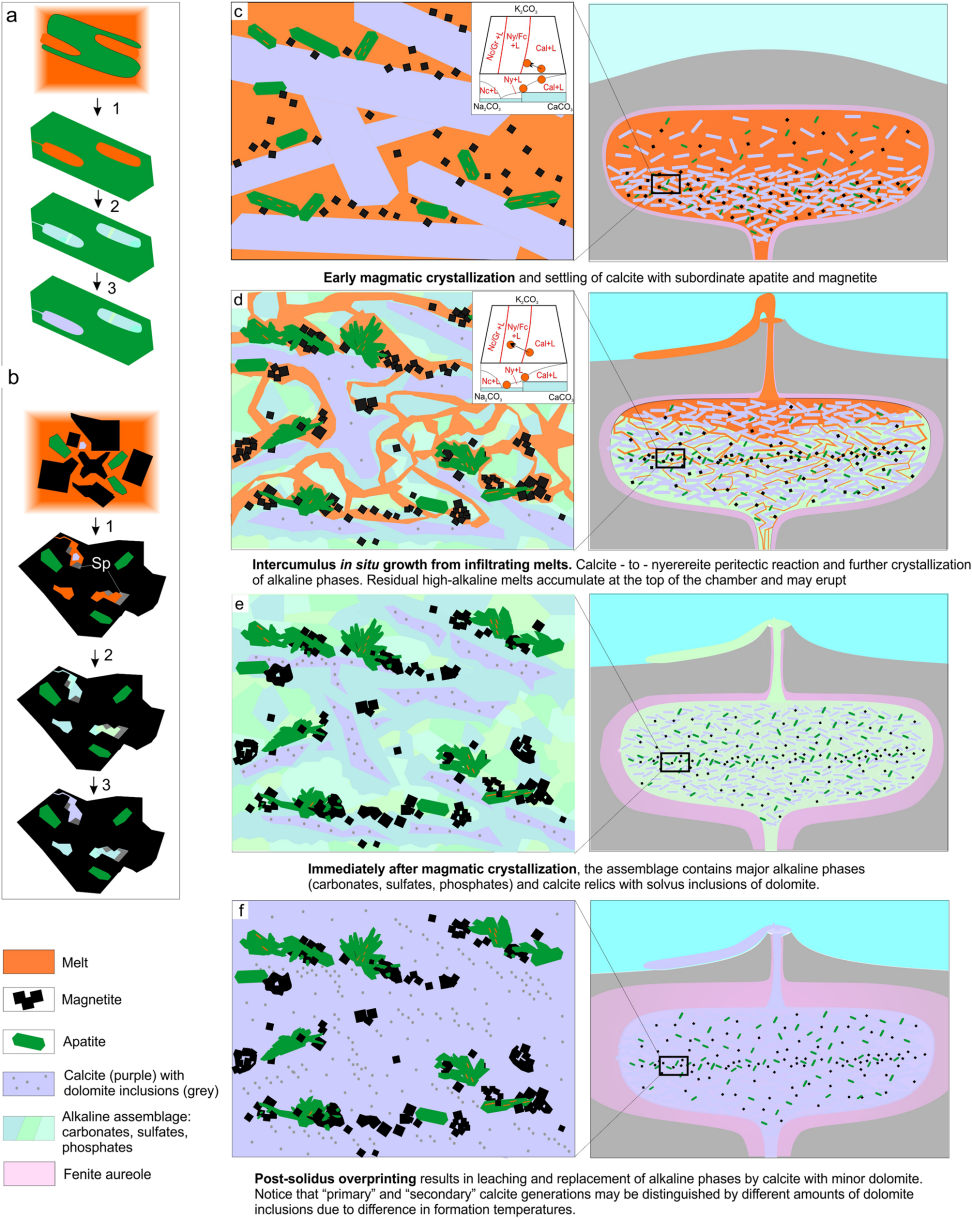
A schematic illustrating the formation of: (**a**)—apatite-I-hosted inclusions; (**b**)—magnetite-hosted inclusions, and (**c**–**f**)—main stages of the evolution of the magmatic system. (**a**, **b**) inset numbered stages are: (1) entrapment; (2) crystallization and (3) overprinting. Note that inclusions which were not isolated completely, are subject to leaching and, ultimately, replacement of alkaline assemblages by calcite and dolomite. Corner insets in (**c**, **d**) show schematic phase diagrams outlining melt evolution paths at given stages (fields outlined from Weidendorfer et al. (2017)^26^ The figure was created using Corel Draw X4 software https://www.coreldraw.com/en/pages/coreldraw-x4/.

Compositions of the experimentally produced melts in apatite-hosted inclusions occupy a relatively narrow range in terms of Na_2_O/CaO-K_2_O/CaO (mass) and CaCO_3_-Na_2_CO_3_-K_2_CO_3_ (molar) relationships (Fig. 4c,d), and are not temperature-dependent (observed across 700–900 °C) (Supplementary Table 6). Given that their mineralogy is typical of other studied inclusions from intrusive and extrusive carbonatites worldwide^14–16,20,23–25^ and the experimentally obtained bulk chemical compositions are similar to entrapped alkaline Ca-carbonatitic melts of the Kerimasi volcano (Fig. 4c,d)^15,16^, we consider these inclusions to represent entrapped and crystallized alkali-rich Ca-carbonatitic melts (Fig. 5a). Since complete melting of crystal phases took place mainly between 700 and 800 °C, whereas the gas bubble did not dissolve or shrink significantly up to high temperatures (1000 °C), we suggest that entrapment of melt + fluid mixture occurred around 700–800 °C. Therefore, further petrological constraints are based on compositions obtained 700 °C and 800 °C, whereas those of 900 °C experiment are used as supplements, statistically corroborating the main dataset.

Magnetite-hosted alkaline assemblages cannot be considered as *bona fide* melt (or melt + fluid) inclusions because melting of alkaline phases occurred mainly in the range 600–700 °C, whereas relatively large grains of spinel, apatite, pyrochlore, baddeleyite remained intact up to 1000 °C, likely implying their solid-state entrapment. However, spinel exsolution, which is characteristic of high-T magnetite^36^, makes it unlikely that fusible alkali-rich carbonates and salts were entrapped as solids also. Rather, hybrid entrapment of alkali-bearing carbonatite melt and crystal phases took place, as has been suggested for a number of different intrusive rocks^43–45^ (Fig. 5b). As melting of all phases, aside from the refractory ones, took place between 600 and 700 °C, we suppose that these hybrid inclusions were entrapped in this temperature range. However, after 600 °C experimental runs, relics of calcite and bradleyite were not uncommon, and the melts contained elevated Na and K compared to the 700–1000 °C melts, which had mostly identical chemical ranges (Supplementary Table 6). Therefore, 600 °C melts were excluded from consideration as most of them were considered “underheated”. We assume compositions of melts, obtained at 700–800 °C (at least their Ca–Na–K relationships) to be proxies for alkaline carbonatite melts, entrapped along with refractory crystal phases during magnetite crystallization, whereas melt compositions obtained from 900 °C and 1000 °C experiments, were considered as supplementary data, as their Ca-Na–K proportions were largely analogous to the lower-temperature melts.

### Lost and found alkalis in intrusive carbonatites

Mineral relationships within the studied rocks provide evidence that their formation involved a range of processes from early magmatic chamber crystallization through to intercumulus growth to post magmatic (subsolidus) modification. Linear textures of mafic and ore minerals (apatite and magnetite in our case) within the groundmass of anhedral mosaic calcite (Fig. 2a,b, Supplementary Figs. 1, 2) were reported in many carbonatites worldwide and have been shown to result from initial (magmatic) alignment of smaller grains of apatite and magnetite along larger tabular phenocrysts of calcite^27^. Distortion and curvature of these initial structures, as well as granulation of calcite, have been attributed to post cumulus or subsolidus recrystallization and plastic deformation^27,46,47^.

In our samples, coarse euhedral apatite-I is assumed to form in free space, i.e. during early magmatic chamber stage of the melt crystallization (Fig. 5a,c). In contrast, the irregular shape of apatite-II (Fig. 2f) and its complex relationships with neighboring phases (Fig. 2e,g) imply in situ growth within a limited space (i.e. syn- or post-cumulus crystal mush) (Fig. 5d). The intricate shape of magnetite grains and their fine-scale intergrowths with other phases (Fig. 2c–e, g–i) also indicate a dominantly in situ growth process. Based on structural observations (Fig. 2h), fine protocrystals of magnetite formed during the early magmatic stage, and were subsequently affected by in situ agglutination and enlargement resulting in intercumulus growth^36,48^ (Fig. 5b,d).

Within this petrographic framework, apatite-I-hosted multiphase inclusions, which are interpreted as primary melt inclusions, are considered to represent melts of the relatively early stage of the carbonatites’ crystallization (Fig. 5a,c). Compositions of these melts were close to those which have been found entrapped within early minerals of the Kerimasi carbonatite volcano, and are proposed to be a possible common parent for carbonatites in general^15,26^.

Textural evidence documented here and previously^44,49,50^ imply that inclusions in magnetite were entrapped mainly during agglutination of protocrystals and growth in the post-cumulus stage (Fig. 5b). Therefore, compositions of melts, obtained at 700–800 °C, are likely representative of intercumulus melts, which were entrapped within these inclusions along with earlier solid phases. More alkaline (particularly, more potassic) compositions of these melts and mineral phases in the unheated inclusions compared to the apatite-I-hosted melt inclusions (Fig. 3i,j; Fig. 4c,d) are coherent with structural evidence that apatite-I crystallization preceded intercumulus growth of magnetite and thus likely reflect evolution of the carbonatitic melt.

Hence, the multiphase inclusions in coexisting apatite-I and post-cumulus magnetite from the Guli and Tagna carbonatites provide empirical evidence of the presence and dominance of alkaline melts in the carbonatite parental environment at both the early and intercumulus stages (Fig. 5c,d) and form a differentiation trend of increasing alkalis (e.g. Fig. 4c,d).

### Alkaline assemblage as a primary feature of the Ca-carbonatite

Plotted on the Na_2_CO_3_-K_2_CO_3_-CaCO_3_ diagram, compositions of the earlier melts (inclusions in apatite-I) are saturated with respect to calcite only (Fig. 4d). In contrast, intercumulus melt compositions (inclusions in magnetite) intersect the peritectic between calcite and the solid solution of nyerereite and fairchildite (hereinafter ‘calcite-nyerereite/fairchildite’ peritectic), and become nyerereite/fairchildite-saturated (Fig. 4d). This implies the replacement of calcite by nyerereite/fairchildite and the crystallization of intercumulus alkaline assemblages (Fig. 5d). In a closed-system scenario, this process should have been limited to a partial replacement of calcite due to the small volume of intercumulus melts. However, abundant in situ crystallized apatite-II and magnetite (whole-rock P and Fe concentrations greatly exceeding the melt inclusions: Supplementary Table 6) imply that the system was open or semi-open at the intercumulus stage. Therefore, it is proposed that during this stage, significant amounts of intercumulus alkaline carbonates and salts, as well as apatite and magnetite, crystallized due to continuous convective infiltration of alkali-rich interstitial melts in accordance with the well-described scenario of “infiltration growth” or “magmatic metasomatism” (Fig. 5d, e)^51–56^.

The interpretation that our apparently alkali-poor sample suite (e.g. Fig. 5f) was initially alkali-rich (e.g. Fig. 5e) requires explanation. This issue has been addressed in a number of studies and it has been argued that chemically unstable alkaline minerals are replaced by more stable calcite (and minor dolomite) during subsolidus transformations. The reality of such process has been proved in examples of the modern Oldoinyo Lengai lavas^57,58^ and widely applied to other extrusive and intrusive carbonatites^13,19,59,60^. However, another hypothesis contends that in most carbonatites, calcite is a primary and dominant magmatic phase, whereas alkaline minerals are rare, particularly in intrusive carbonatites of cumulative origin^25,61^. As our data imply that: (1) alkaline phases must have abundantly crystallized during at least the intercumulus stage of formation and (2) present granular and anhedral calcite is not *bona fide* primary magmatic, but rather formed during near-solidus or subsolidus recrystallization^27,46^, the replacement of the alkaline minerals by calcite (and minor dolomite) provides a feasible explanation for the studied cases. High plasticity and extensive recrystallization of calcite at depths did not allow for development of significant porosity of the present rocks, which has been claimed to be inevitable during modification of extrusive carbonatites at atmospheric pressure^61^. These post-magmatic transformations could also be responsible for chemical re-equilibration of the minerals and, thus overprint any chemical evidence for “primary” and “secondary” generations (Fig. 5f). However, we have noticed that inner parts of large calcite domains (formerly early phenocrysts of calcite according to Zhabin (1971)^27^) are slightly richer in dolomite inclusions than calcite in apatite- and magnetite-rich domains (formerly interstitial spaces^27^) (Supplementary Fig. 1). This may serve as indirect evidence for two generations of calcite and support that the “interstitial” calcite was formed during a lower temperature process (e.g. due to near-solidus or subsolidus replacement of the alkaline carbonates) (Fig. 5e,f).

As a result, we suggest that the primary magmatic assemblage of the studied rocks contained major alkaline carbonates and salts (Fig. 5e). Depletion in alkalis of the present assemblage is best explained by near-solidus or subsolidus replacement of unstable alkaline carbonates, sulfates and salts by calcite and minor dolomite (which likely originated initially by exsolution from high-T calcite) (Fig. 5f). Simultaneous or subsequent recrystallization of the precursor rocks resulted in compaction, development of metamorphic textures and chemical re-equilibration of the minerals.

### Intercumulus melts—a missing piece in the Oldoinyo Lengai puzzle

Both empirical and experimental studies lead us to propose that moderately alkaline Ca-carbonatite melts (e.g. melt inclusions from the Kerimasi volcano) are a common parent for both Ca- and Na-carbonatites^15,16,26^. However, in the Na_2_CO_3_-K_2_CO_3_-CaCO_3_ system, there is a significant gap between the Kerimasi volcano inclusions (calcite-saturated) and the Oldoinyo Lengai (nyerereite- and fairchildite-saturated) compositions (Fig. 4c,d). Theoretically, at the calcite-nyerereite/fairchildite peritectic, the composition of the melt remains buffered by calcite and should not evolve further until calcite has been exhausted. Therefore, this peritectic might be a significant barrier which inhibits differentiation of an alkaline carbonatitic melt and precludes transition of the melt’s composition from calcite- to nyerereite-saturated fields (Fig. 4c,d). However, our data and observations documented here (Fig. 4c,d, Supplementary Table 6), clearly show that the compositions of the intercumulus melts from the Guli and Tagna carbonatites intersect the calcite-nyerereite/fairchildite peritectic, linking the Kerimasi and Oldoinyo Lengai compositions. Based on this evidence, we argue that Na-carbonatitic melts, similar to those of the Oldoinyo Lengai, may originate during fractionation of a moderately alkaline Ca-carbonatitic melt and provide, therefore, the “missing piece” in the puzzle of the Na-carbonatites origin. In an assumption that the effusive Na-carbonatites are discharged intercumulus (residual) melts, the uniqueness of the Oldoinyo Lengai case may be explained by juxtaposition of the following required conditions: (1) alkalis were not lost at the earlier stage, (2) the starting melt was sufficiently alkaline or was effectively isolated from cumulus calcite to surpass the calcite-nyerereite/fairchildite peritectic, and (3) the residual melt retained alkalis during upwelling.

## Conclusions

Alkaline carbonatitic melts were parental for the alkali-poor intrusive Ca-carbonatites of the Guli and Tagna massifs (Siberia, Russia). The alkalis were not lost at early magmatic stages, and their concentrations in the late intercumulus melts approached high values, similar to those of the Oldoinyo Lengai Na-carbonatitic lavas. The data obtained provide the first direct empirical evidence that (1) a primary magmatic assemblage of intrusive carbonatites may be substantially alkaline and (2) Na-carbonatites similar to the Oldoinyo Lengai lavas may represent discharged residual melts derived from an alkali-bearing Ca-carbonatitic ‘common parent’ via fractional crystallization. Given this evidence, we suggest that many occurrences of alkali-poor intrusive carbonatites worldwide initially contained high concentrations of Na and K and were accompanied by alkaline carbonatitic volcanism (similar to the Oldoinyo Lengai case). Mechanisms of the alkalis’ loss from the primary magmatic assemblages remain disputable and may involve processes of deuteric (or other hydrothermal) replacement of alkaline carbonates and salts by calcite and dolomite.

## Supplementary Information

Supplementary Information 1.

Supplementary Information 2.

Supplementary Information 3.

Supplementary Information 4.

Supplementary Information 5.

Supplementary Information 6.

Supplementary Information 7.

Supplementary Information 8.

Supplementary Information 9.

Supplementary Information 10.

Supplementary Information 11.
