# Supplementary material for: Origin of alkali-rich volcanic and alkali-poor intrusive carbonatites from a common parental magma
Source: Sci Rep. 2021 Sep 2;11:17627. doi: 10.1038/s41598-021-97014-y (PMC8413459; doi:10.1038/s41598-021-97014-y)
Supplement: Supplementary file 1 — Supplementary Information 1. [file 41598_2021_97014_MOESM1_ESM.docx]

**CAPTIONS OF THE SUPPLEMENTARY MATERIALS**

**Supplementary Figure 1.** A high-resolution BSE image of a polished thin section of the Guli carbonatite. Outlined is a small part of a calcite-rich domain in contact with the apatite-magnetite-rich one (see text for the details). Abbreviations: Ap – apatite, Ol – olivine, Cal+Dol – calcite-dolomite groundmass aggregate, Mag – magnetite. The original BSE-photo has been processed using Corel Draw X4 software https://www.coreldraw.com/en/pages/coreldraw-x4/

**Supplementary Figure 2.** A high-resolution BSE image of a polished section of the Tagna carbonatite. Abbreviations: Ap – apatite, Ol – olivine, Cal+Dol – calcite-dolomite groundmass aggregate, Mag – magnetite. The original BSE-photo has been processed using Corel Draw X4 software https://www.coreldraw.com/en/pages/coreldraw-x4/

**Supplementary Figure 3.** Phase transformations in an apatite-I-hosted alkaline multiphase inclusion during heating experiments. Temperatures, to which the snapshots correspond, are given in the upper right corner. Note that as melting of sulfates begins between 300 and 400 oC, carbonates become progressively darker from 400 to 650 oC, whereas at 720 oC the inclusion decrepitates and leaks. The figure has been created by compilation of optical photomicrographs using Corel Draw X4 software https://www.coreldraw.com/en/pages/coreldraw-x4/.

**Supplementary Figure 4.** Phase transformations in an apatite-I-hosted alkaline multiphase inclusion during heating experiments. Temperatures are given in the upper left corner. The figure has been created by compilation of optical photomicrographs using Corel Draw X4 software https://www.coreldraw.com/en/pages/coreldraw-x4/.

**Supplementary Figure 5.** SEM BSE images of inclusions after heating and quenching experiments. (a) Apatite-hosted inclusion, Tagna carbonatite 800 oC; (b) Magnetite-hosted inclusion with nyerereite (Ny) “spinifex” and brucite (Br), Tagna carbonatite, 800 oC; (c) Magnetite-hosted inclusion with former melt pools, formed by alkaline carbonate aggregate and crystals of refractory spinel (Sp), brucite (Br), geikielite (Gkl), Mg-Al hydrosilicate (Sil) and a small unmolten relic of calcite, Guli carbonatite, 600 oC; (d) Magnetite-hosted inclusion with refractory pyrochlore (Pcl), spinel (Sp) and newly formed quench crystals of apatite (Ap), Guli carbonatite, 700 oC. The figure has been created by compilation of BSE photos using Corel Draw X4 software https://www.coreldraw.com/en/pages/coreldraw-x4/.

**Supplementary Figure 6.** Custom designed experimental setup with a pipe furnace developed for instant quenching. (a) General view with temperature control technique; (c) Top view of the pipe furnace. The figure has been created by processing of a camera photo using Corel Draw X4 software https://www.coreldraw.com/en/pages/coreldraw-x4/.

**Supplementary Table 1.** Compositions of olivine from the Guli carbonatite (EPMA WDS data)

**Supplementary Table 2.** Compositions of apatite from the Guli carbonatite (EPMA WDS data)

**Supplementary Table 3.** Compositions of calcite from the Guli carbonatite (SEM EDS data)

**Supplementary Table 4.** Mineral phases in alkaline multiphase inclusions and their occurrence distribution (%). Mag-hosted inclusions from the Tagna carbonatites were scarce and are not included into the list. They comprise nyerereite/shortite and bradleyite.

**Supplementary Table 5.** Compositions of the alkaline carbonates from the multiphase inclusions (EDS data, wt. %)

**Supplementary Table 6.** Compositions of melts in heated and quenched inclusions from the Guli and Tagna carbonatites (EDS data, wt. %)
