# Supplementary figures and images for: Origin of alkali-rich volcanic and alkali-poor intrusive carbonatites from a common parental magma
Source: Sci Rep. 2021 Sep 2;11:17627. doi: 10.1038/s41598-021-97014-y (PMC8413459; doi:10.1038/s41598-021-97014-y)

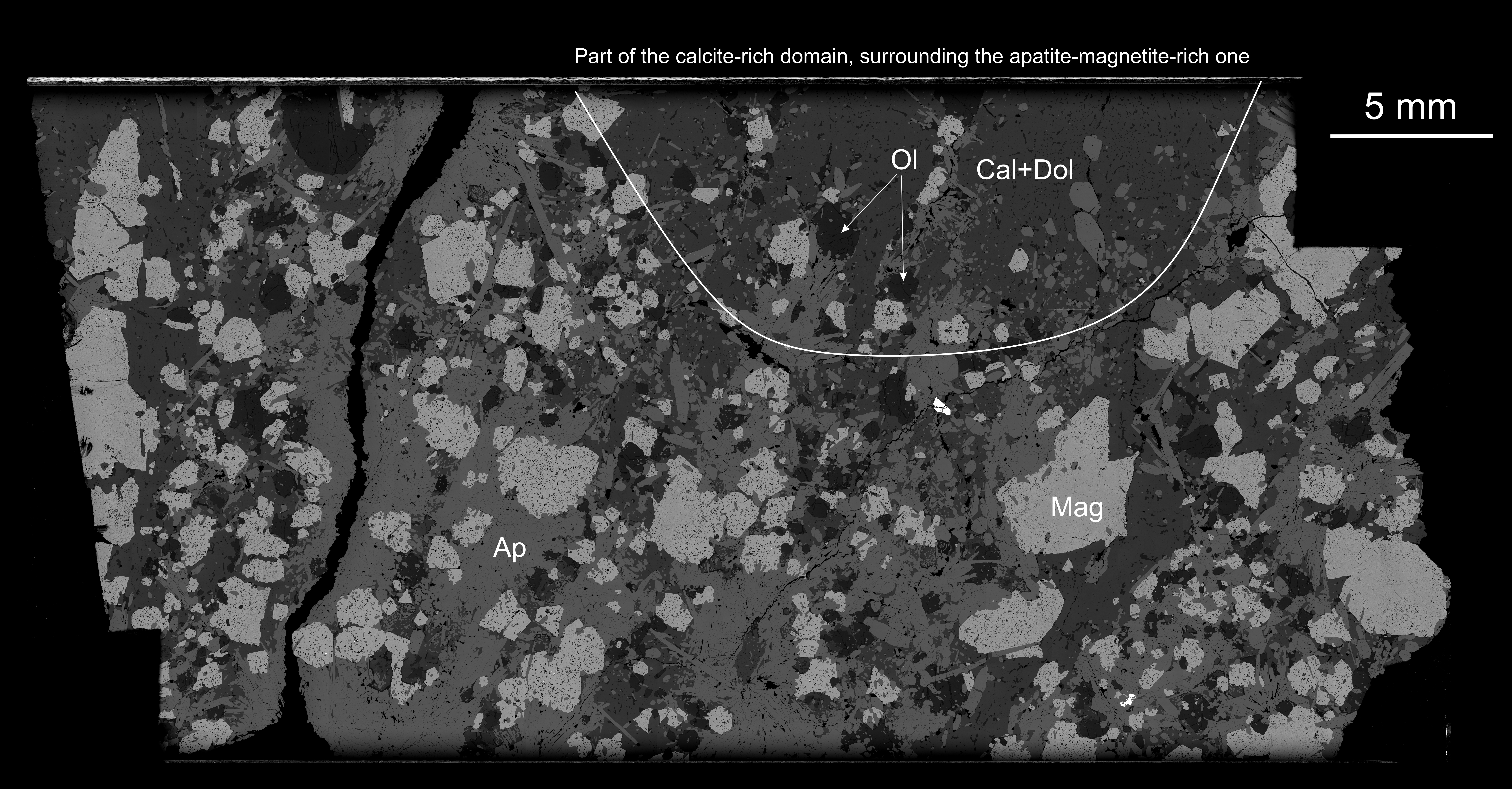

Supplement: Supplementary file 2 — Supplementary Information 2. [file 41598_2021_97014_MOESM2_ESM.jpg]

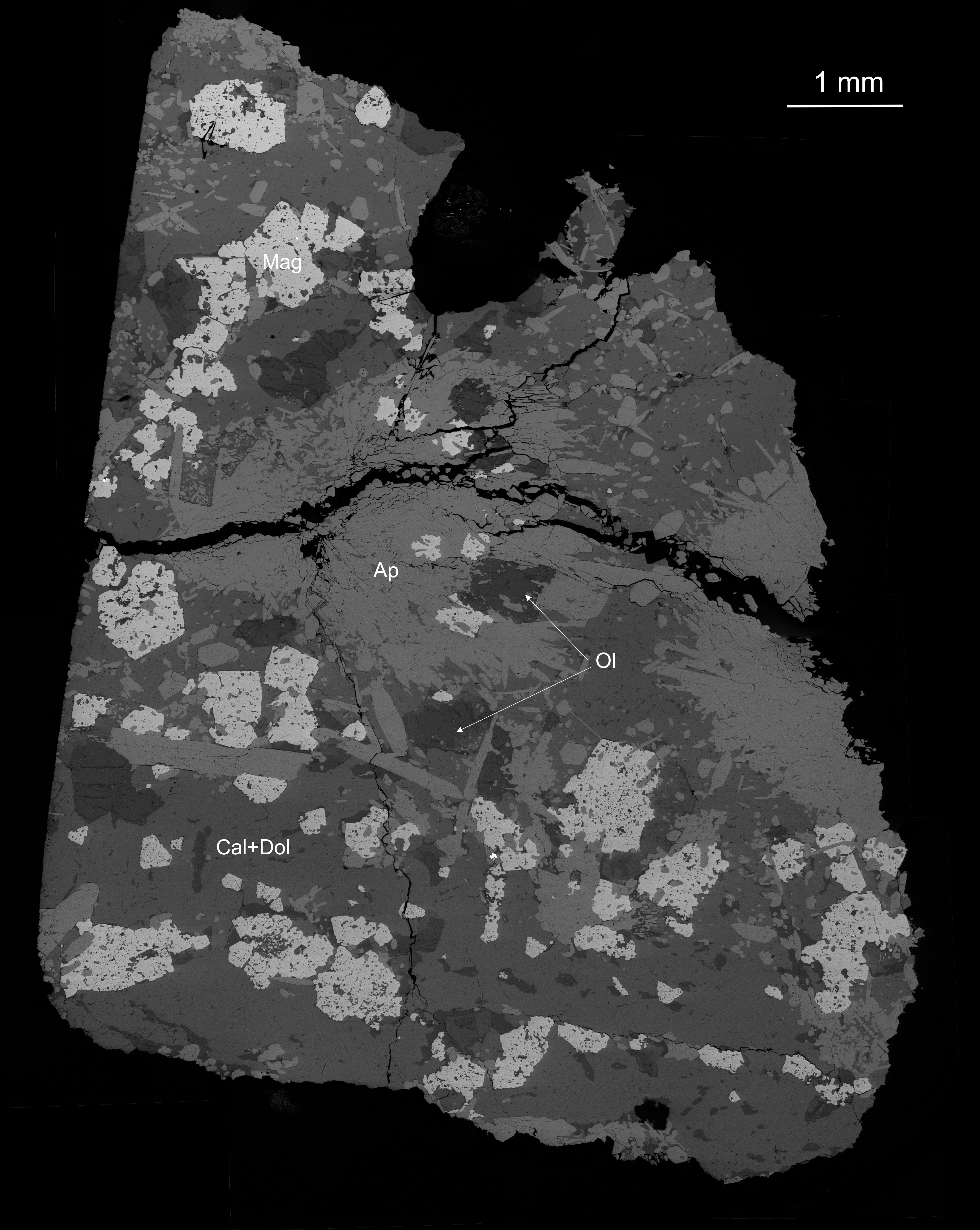

Supplement: Supplementary file 3 — Supplementary Information 3. [file 41598_2021_97014_MOESM3_ESM.jpg]

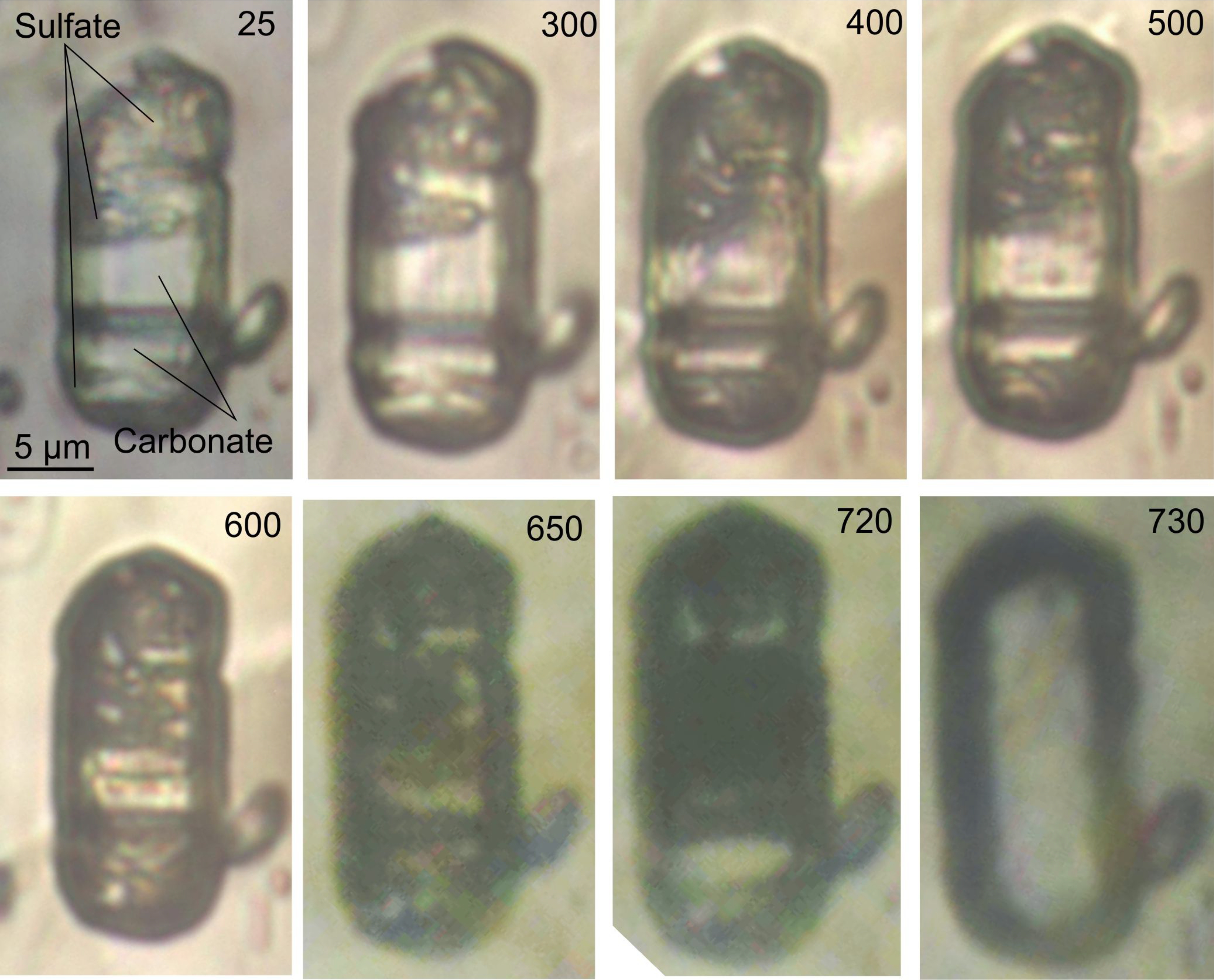

Supplement: Supplementary file 4 — Supplementary Information 4. [file 41598_2021_97014_MOESM4_ESM.jpg]

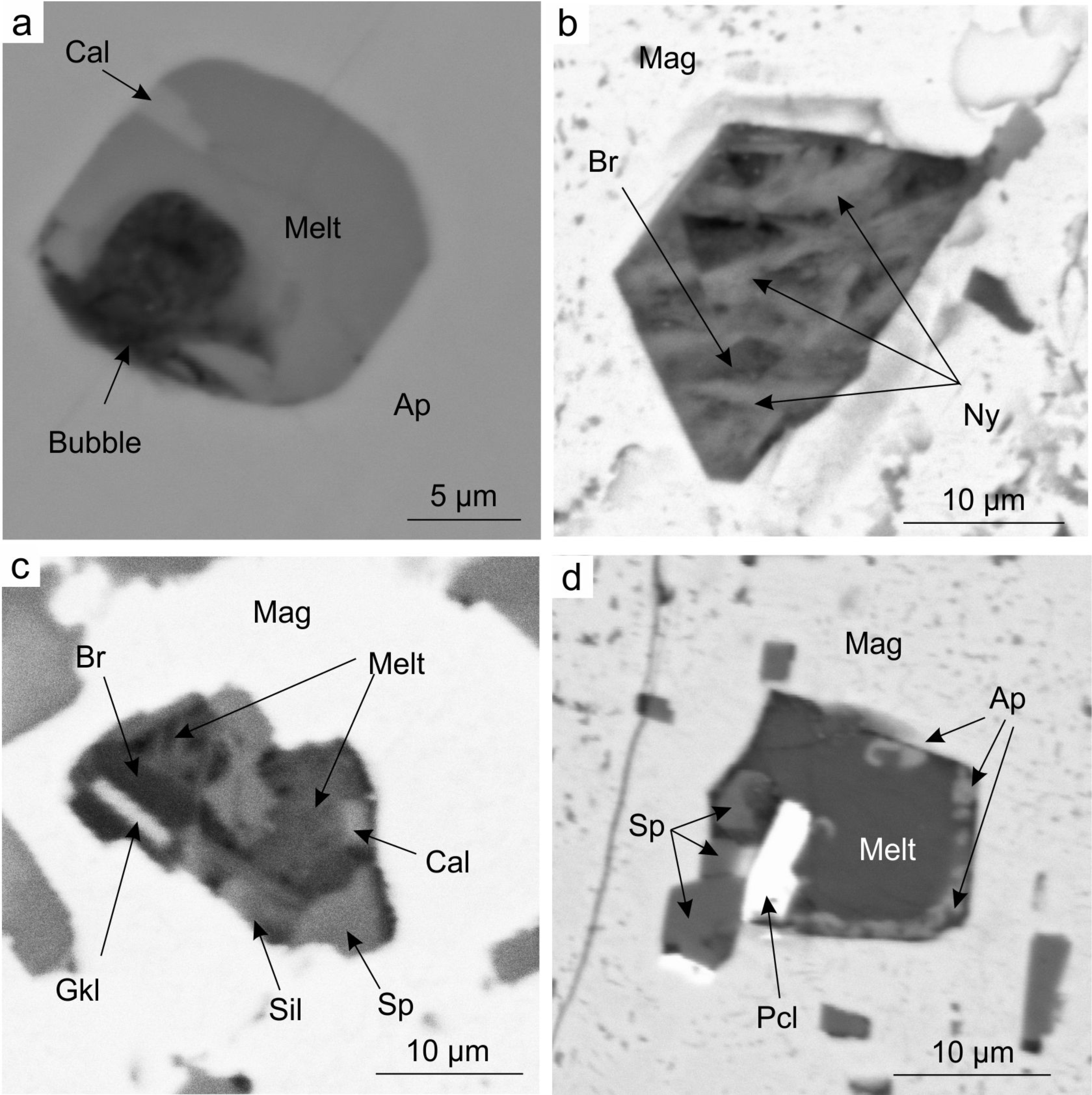

Supplement: Supplementary file 6 — Supplementary Information 6. [file 41598_2021_97014_MOESM6_ESM.jpg]

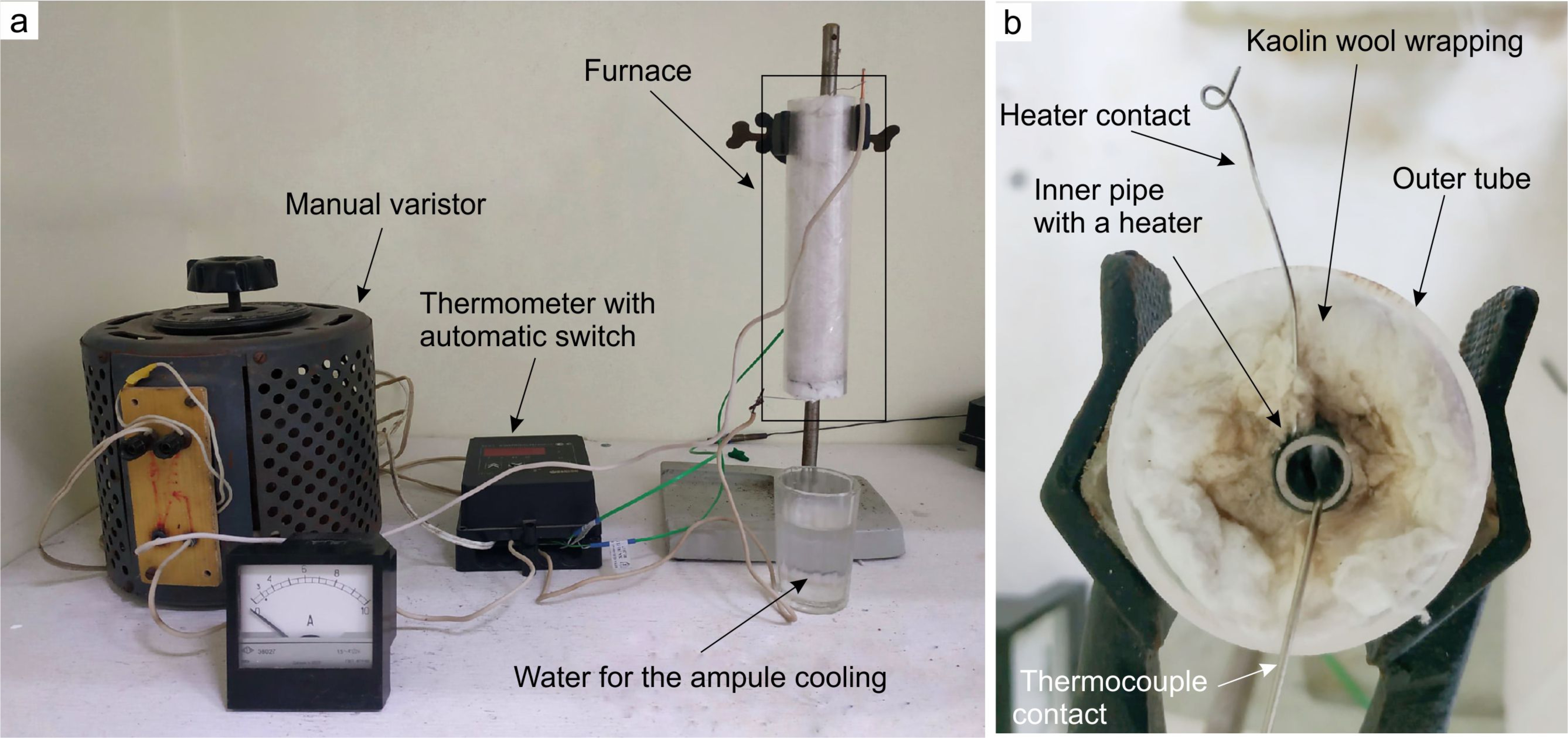

Supplement: Supplementary file 7 — Supplementary Information 7. [file 41598_2021_97014_MOESM7_ESM.jpg]
