# Supplementary material for: Origin of alkali-rich volcanic and alkali-poor intrusive carbonatites from a common parental magma
Source: Sci Rep. 2021 Sep 2;11:17627. doi: 10.1038/s41598-021-97014-y (PMC8413459; doi:10.1038/s41598-021-97014-y)
Supplement: Supplementary file 9 — Supplementary Information 9. [file 41598_2021_97014_MOESM9_ESM.pdf]

| | | Ideal formula | Ap-hosted, Guli | Mag-hosted, Guli | Ap-hosted, Tagna |
|---|---|---|---|---|---|
| Carbonates | Ankerite | $Ca(Fe,Mg)(CO_3)_2$ | 8 | - | |
| | Burbankite | $(Na,Ca)_3(Sr,Ba,Ce)_3(CO_3)_5$ | 32 | 2 | 30 |
| | Calcite | $CaCO_3$ | 38 | 16 | 1 |
| | Dolomite | $CaMg(CO_3)_2$ | 3 | 27 | 1 |
| | Eitelite | $Na_2Mg(CO_3)_2$ | - | 24 | - |
| | Hydrotalcite | $(Mg,Fe)_6(Al,Fe)_2(CO_3)(OH)_{16}\cdot 4H_2O$ | 2 | 31 | |
| | Magnesite | $MgCO_3$ | - | 2 | |
| | Northupite | $Na_3Mg(CO_3)_2Cl$ | - | 7 | |
| | Shortite/nyerereite | $(Na,K)_2Ca(CO_3)_2$ / $(Na,K)_2Ca_2(CO3)_3$ | 75 | 47 | 85 |
| | Siderite | $FeCO_3$ | - | 9 | |
| | Smithsonite | $ZnCO_3$ | 7 | - | |
| | Trona | $Na_3H(CO_3)_2\cdot 2H_2O$ | 3 | - | |
| | Witherite | $BaCO_3$ | - | 4 | |
| Sulfates | K-bearing mixed: arcanite, aphthitalite, görgeyite, bubnovaite | $K_2SO_4$<br>$(K,Na)_3Na(SO_4)_2$<br>$K_2Ca_5(SO_4)_6 H_2O$<br>$K_2Na_8Ca(SO_4)_6$ | 12 | 9 | 25 |
| | Celestine | $SrSO_4$ | - | 2 | |
| | Celestine-(Ba) | $(Sr,Ba)SO_4$ | - | 2 | |
| | Thenardite/glauberite | $Na_2SO_4$ / $Na_2Ca(SO_4)_2$ | 38 | 4 | 35 |
| Phosphates | Apatite | $Ca_5(PO_4)_3(Cl,F,OH)$ | - | 16 | - |
| | Bradleyite | $Na_3Mg(CO_3)(PO_4)$ | 15 | 22 | 15 |
| | Monazite-Th | $Th(PO_4)$ | 3 | - | |
| Halides | Halite | $NaCl$ | 5 | 7 | 15 |
| | Sylvite | $KCl$ | 2 | 4 | |
| Silicates | Clinochlore | $Mg_5Al(AlSi_3O_{10})(OH)_8$ | 3 | - | |
| | Glagolevite | $Na(Mg,Al)_6(AlSi_3O_{10})(OH,O)_8$ | 17 | 4 | 5 |
| | Serpentine | $Mg_3[Si_2O_5](OH)_4$ | - | 2 | |
| | Titanite | $CaTi(SiO_4)O$ | - | 2 | |
| Oxides/hydroxides | Aeschynite | $(Ce,Ca,Fe,Th)(Ti,Nb)_2(O,OH)_6$ | - | 4 | |
| | Baddeleyite | $ZrO_2$ | - | 9 | |
| | Brucite | $Mg(OH)_2$ | - | 36 | |
| | Cr-spinel | $CrAl_2O_4$ | 2 | - | |
| | Fersmite | $(Ca,Ce,Na)(Nb,Ta,Ti)_2(O,OH,F)_6$ | - | 13 | |
| | Geikielite | $MgTiO_3$ | - | 9 | |
| | Ilmenite | $FeTiO_3$ | - | 2 | |
| | Magnetite | $FeFe_2O_4$ | 2 | - | |
| | Rutile | $TiO_2$ | - | 4 | |
| | Spinel | $MgAl_2O_4$ | - | 80 | |
| | Thorianite | $ThO_2$ | 2 | - | |
| | Zirconolite | $CaZrTi_2O_7$ | - | 2 | |
| Sulfides | Chalcopyrite | $CuFeS_2$ | 3 | - | |
| | Pentlandite | $(FeNi)_9S_8$ | 2 | - | |
| | Sphalerite | $ZnS$ | - | 2 | |
| | Stibnite | $Sb_2S_3$ | 2 | - | |
